# Tracking the early spatio-temporal dynamics of phytoplasma multiplication within its leafhopper vector

**DOI:** 10.1099/mic.0.001700

**Published:** 2026-04-29

**Authors:** Valeria Trivellone, Francesca Canuto, Giulia Lucetti, Christopher H. Dietrich, Luciana Galetto, Cristina Marzachì

**Affiliations:** 1 Illinois Natural History Survey, Prairie Research Institute, University of Illinois at Urbana Champaign, 1816 South Oak Street, Champaign 61820, Illinois, USA; 2 Institute for Sustainable Plant Protection-IPSP, National Research Council of Italy-CNR, Strada delle Cacce 73, 10135, Turin, Italy

**Keywords:** acquisition access period, *Candidatus*, carrying capacity, inoculation, insect, not-yet-cultivable bacteria, phytoplasma load

## Abstract

Transmission of phytoplasmas, recalcitrant, yet-to-be-cultivated bacteria, by insect vectors depends on acquisition and subsequent pathogen multiplication within the insect body. However, the influence of acquisition duration on early colonization dynamics remains poorly understood. This study clarifies the spatio-temporal patterns of *Flavescence dorée* phytoplasma (16SrV-D) multiplication in its leafhopper vector *Euscelidius variegatus* during the early stages of infection when acquisition access period (AAP) is short. Insects were exposed to two acquisition conditions: a short 4-h AAP, simulating incidental feeding, and a 2-day AAP, considered the minimum threshold for effective acquisition. Phytoplasma load was quantified in the head and body over time, and these data were integrated with published data covering longer AAPs (7–14 days) and post-acquisition periods (up to 42 days). A Bayesian hurdle-lognormal model was used to describe temporal dynamics and estimate pathogen multiplication rates across studies. Very short AAPs led to significantly lower phytoplasma loads after an 8-day latent period compared to 2-day AAPs, highlighting the importance of feeding duration for efficient colonization. Model predictions indicated that phytoplasma load after short AAPs increases gradually and follows similar temporal trends to those observed for longer AAPs, but remained consistently lower across the 40-day post-acquisition period. Nevertheless, empirical and model-based estimates suggest that even brief feeding events, particularly 2 days, can yield pathogen loads approaching the minimum transmission threshold (10³–10⁴ genome units ng⁻¹ insect DNA) in some individuals, as suggested by upper CI bounds, with potential to sustain infection and facilitate transmission. These findings shed light on how acquisition duration shapes early phytoplasma dynamics in vectors and offer insights into transmission risk under natural conditions where incidental feeding events may occur.

## Graphical Abstract

Spatio-temporal dynamics of phytoplasma multiplication in a leafhopper following 4-h and 2-day acquisition periods, integrating experimental and published data to model load trajectories and estimate minimum thresholds and carrying capacity.

## Introduction

Vector-borne pathogens rely on mobile insect vectors to spread among definitive hosts. Vector–pathogen associations range from non-circulative, non-propagative interactions to fully circulative, propagative cycles. In all cases, pathogens exploit the vector’s cellular and molecular machinery to some extent, and in circulative, propagative systems, this exploitation extends to active multiplication within the vector, ultimately transforming it into an infectious life-long carrier, capable of transmitting the pathogen to new hosts [1,2]. To characterize the dynamics of circulative, propagative transmission strategies, experimental approaches are essential for describing pathogen acquisition and multiplication within the vector. In the case of plant pathogens, one key experimental parameter is the acquisition access period (AAP), defined as the time during which non-infected insect vectors are exposed to infected plants and allowed to feed on them [1]. Another important parameter is the latent period (LP), which is defined as the time, post-acquisition, needed for the vector to become infectious or the time needed for a pathogen to reach the salivary glands [3]. For sap-feeding vectors, the term ‘feeding event’ refers to the specific period during which an insect feeds on the phloem, xylem or associated cell contents without interruption. The electrical penetration graph technique allows researchers to determine which tissue the insect feeds on and for how long [4]. For example, Ripamonti *et al.* [5] measured the longest phloem ingestion, a single feeding event of 4 h, by allowing the leafhopper *Scaphoideus titanus* (Ball), the main vector of Flavescence dorée (FD) phytoplasma of grapevine, to feed on the most palatable grapevine variety. The number of phytoplasma cells acquired during a given AAP depends not only on its total duration but also on the quality and length of individual feeding events, which may vary in both experimental settings and in nature, based on biological and environmental factors such as insect sex, developmental stage, host plant identity and climatic conditions [5,7]. In the same way, the LP may vary from a minimum of 7–28 days, depending on the specific host–pathogen association, the length of the AAP and the initial pathogen load [3,7, 8]. Previous empirical studies have provided insight into the minimum AAP required for successful phytoplasma acquisition while optimizing their transmission experiments. Seminal studies have correlated the duration of AAP with transmission rate. For example, Chiykowski [9,10] found that the infection rate varied from 1.5 to 85% for AAPs ranging from 0.5 h to 14 days, respectively, using the *Macrosteles quadrilineatus* (Forbes) – ‘*Candidatus* (*Ca*.) Phytoplasma (P.) asteris’ system. More recently, Rashidi *et al.* [11] reported that for a strain of the same phytoplasma species (Chrysanthemum yellows phytoplasma, CYp), a minimum AAP of 4 h was necessary to observe the first phytoplasma-positive leafhoppers, with 20 and 40% infection rates in *Euscelidius variegatus* (Kirschbaum) and *Macrosteles quadripunctulatus* (Kirschbaum), respectively, after the LP. With an AAP of 24 h, the infection rates increased to 47 and 75%, respectively. Galetto *et al.* [12], working again with the *E. variegatus*–CYp system, showed that after an AAP of 2 days, all 85 tested individuals were infected, with phytoplasma loads ranging from 1.83×10⁻⁶ to 2.50×10⁻¹ CYp 16SrRNA/insect glyceraldehyde-3-phosphate dehydrogenase (GAPDH) transcript. Another study using a different vector-phytoplasma system [*Cacopsylla melanoneura* (Förster) – ‘*Ca*. P. mali’] reported similar results even though the vector’s life is much longer than that of the cicadellids considered in the above-described associations [13]. In the *Matsumuratettix hiroglyphicus* (Matsumura) – sugarcane white leaf phytoplasma system, the phytoplasma load in adult insects increased with time after 15 min of AAP and reached a plateau 6–12 h later [7]. Together, these findings suggest that even short acquisition periods may suffice for successful infection, but the resulting phytoplasma load, and thus transmission potential, likely depends on both AAP length and subsequent systemic colonization of the vector’s body. In this context, focusing on the ‘*Ca*. P. asteris’ system [14,17] or the FDp system [11,18,21], other studies have reported the dynamics of phytoplasma multiplication in different organs of insect vectors. All these studies showed an increase in phytoplasma load over time; however, the tested AAP was fixed at 7, 10 or 14 days, with measurements taken post-acquisition starting after 7 days. Notably, only one previous study [18] investigated early infection dynamics by quantifying FD phytoplasma load daily during the first 7 days post-acquisition (dpa, so-called LP).

Our review of the literature revealed a gap in knowledge regarding the early dynamics of phytoplasma multiplication following short AAPs. Several fundamental questions remain unanswered, and addressing them could improve understanding of how phytoplasmas colonize insect vectors under the challenging and variable conditions found in natural settings. One important but rarely addressed natural scenario is incidental acquisition, in which potential vectors probe an infected plant for a short period of time, acquiring a small initial pathogen load. Such events may occur in the field due to incidental or temporary host-plant shifts, yet their contribution to epidemiology remains poorly understood. While many studies have quantified phytoplasma load after the LP, data on early post-acquisition phases are scarce.

For vector-borne pathogens, the minimum pathogen load in the insect vector required to enable transmission is a key epidemiological parameter [22,23]. Using three different patho-systems, the lowest phytoplasma load required to overcome the salivary barrier and allow phytoplasma transmission has been shown to range from 10^3^ to 10^4^ genome units (GU) ng⁻¹ insect DNA [18,24, 25]. In basic ecological research, the concept of carrying capacity (the maximum number of individuals an environment can support) appears to be intrinsically dynamic, depending on several factors. For example, the environment itself may change over time [26]. In the case of obligate parasitic bacteria such as phytoplasmas, the vector’s body serves as the ‘environment’, and population growth is constrained by the intracellular microhabitat requirements. Empirical data from different Auchenorrhynchan-phytoplasma models suggest that this carrying capacity is ~10⁵ GU ng⁻¹ insect DNA (e.g. 21), although this value can be influenced by factors such as the insect’s lifespan. Incorporating this concept into our study provides a useful reference point to assess whether short AAPs can lead to pathogen loads approaching the maximum observed capacity, as well as to estimate the average phytoplasma multiplication rate.

This study aims to clarify the spatio-temporal dynamics of phytoplasma multiplication during the early stages of insect host colonization. Insect vectors were exposed to two acquisition conditions: (i) a short 4-h AAP [27], which simulates an incidental acquisition event, and (ii) a 2-day AAP, widely considered the minimum threshold for effective acquisition [12]. Specifically, we set up experiments to characterize phytoplasma accumulation over time in the body and head of the vector, two compartments representing key physical and molecular barriers to phytoplasma multiplication and transmission, and to assess the effect of different acquisition durations on early colonization and establishment. To contextualize our findings, we integrated data from the literature representing longer acquisition durations (7–14 days) and longer post-acquisition periods (up to 42 days) on the *E. variegatus*–FDp patho-system. This allowed us to compare phytoplasma load trajectories under minimal versus optimal acquisition conditions. We also calculated empirical multiplication rates across studies to validate model-derived predictions and to explore whether short AAPs can sustain phytoplasma proliferation over the vector’s lifespan and approach the known carrying capacity (10⁵ GU ng⁻¹ insect DNA; 8, 18, 19, 20, 21).

We addressed the following research questions.

Q1 – Does a short-term feeding event (4 h, 4-h AAP) lead to a similar spatio-temporal phytoplasma load compared to the minimum acquisition period (2 days, 2-day AAP) known to support effective colonization?

Q2 – How are phytoplasma load trajectories predicted to evolve over time following short (4 h) versus longer (2 days) acquisition periods, as observed in empirical studies up to 42 days?

Q3 – Does pathogen load increase sufficiently over time in different acquisition treatments to approach the threshold titre for vector competence (10^3^–10^4^ GU ng⁻¹ insect DNA) and carrying capacity (10⁵ GU ng⁻¹ insect DNA), or do shorter AAPs constrain long-term colonization potential?

To investigate this, we employed a well-established experimental system: *E. variegatus* and FD phytoplasma, strain 16SrV-D (hereafter FD-Dp). This model is recognized for its high experimental efficiency, with previous studies showing that *E. variegatus* can acquire FD-Dp from and transmit to *Vicia faba* with a minimum incubation time of 25 days, compared to *Euscelis incisa* (Kirschbaum) (40 days), with higher infection rates after a 2-week AAP [28,30].

## Methods

### Biological model and experimental setting

Specimens of *E. variegatus* were obtained from phytoplasma-free colonies at the IPSP-CNR (Turin, Italy) and reared on *Avena sativa* (oat) in climate-controlled chambers at 20–25 °C with a 16:8 h photoperiod. FD-Dp was routinely maintained under controlled conditions through sequential transmissions from and to *V. faba* (broad bean) plants by the experimental vector *E. variegatus*.

In this experiment, two AAPs were tested based on data available in the literature (Material S1, available in the online Supplementary Material): 4 h, representing a very short contact event, and 2 days, representing the minimum most effective AAP time. Before each AAP, all individuals were starved for 2–4 h.

To account for mortality and other unexpected events, a total of 280 individuals (140 males and 140 females) from phytoplasma-free insect colonies were used for each experiment (4-h and 2-day AAP). The insects completed each experimental AAP treatment in two different cages on two FD-Dp-infected broad bean plants with similar phytoplasma load (ranging from 3.75E+02 to 5.44E+03 GU ng⁻¹ plant DNA, Material S2). After the AAP, the plants were removed and replaced with three phytoplasma-free oat plants to feed the insects and allow phytoplasma multiplication. Starting from the end of the AAP, 10 independent replicates (5 males and 5 females) were collected from each cage (4 h and 2 days) every 8 and 16 h alternatively for 8 days (for a total of 17 sampling periods). A total of 340 individuals were collected (170 from the 4-h cage and 170 from the 2-day cage). Each individual was dissected into two subsamples (head and body) on a Petri dish containing dry ice to preserve tissue integrity and placed in a 1.5 ml tube, for a total of 720 subsamples. The subsamples were preserved at −80 °C prior to nucleic acid extraction, detection and quantification.

### Extraction of nucleic acids and phytoplasma quantification

To monitor changes in phytoplasma load over time, individuals were analysed at nine time points: immediately post-acquisition (0 h post-acquisition, hpa), and subsequently at 48, 96, 136, 144, 160, 168, 184, 192 hpa, equivalent to 0, 2, 4, 5.7, 6, 6.7, 7, 7.7 and 8 dpa. The rationale was to identify the onset of exponential phytoplasma growth and increase the analysis frequency from that point (i.e. after 5 days). A total of 352 head/body subsamples were analysed (corresponding to 176 individuals: 10 individuals per period×9 periods×2 treatments, with 6 specimens lost during the last period).

Starting from the assumption that we were working with potential low phytoplasma loads, we considered RNA quantification to improve diagnosis and detection. However, RNA-based measurements are not directly comparable to the data available in the literature, which are typically expressed as GU ng⁻¹ of insect DNA. Therefore, we chose to extract total nucleic acids (DNA and RNA) to ensure that RNA would be available for quantification if genomic DNA yields proved insufficient for detection.

The extraction method was selected by comparing the traditional cetyltrimethylammonium bromide (CTAB) DNA extraction procedure [31] (protocol 1, Material S3) with a total RNA extraction kit (Spectrum^™^ Plant Total RNA Kit, Sigma-Aldrich) used without the optional DNase step (protocol 2, Material S3). Since the DNA yields from the two protocols were comparable (Table S1), we selected protocol 2 to simultaneously obtain both DNA and RNA. Briefly, frozen subsamples were transferred into liquid nitrogen and crushed with a micropestle. Total DNA and RNA were co-extracted using Spectrum^™^ Plant Total RNA Kit, slightly modified as described in Material S3. Extracted nucleic acids were quantified using a Nanodrop ND-1000 spectrophotometer (Thermo Fisher Scientific). Samples from the body were diluted 1:10, while those from heads were kept undiluted.

Quantitative PCR (qPCR) was carried out using a modified [31] method, with primers targeting phytoplasma *map* gene combined with SYBR Green chemistry (iTaq Universal SYBR Green Supermix, Bio-Rad) and standard curve of plasmid-containing target FD-Dp amplicons [32] and expressed as GU ng⁻¹ insect DNA, taking into account dilution of body samples.

DNA from the infected plants used during the AAP was extracted with CTAB DNA extraction procedure (Material S2) [31] and quantified with the same procedure.

### Statistical approach

To compare phytoplasma load between sexes as well as between head and body tissues across acquisition treatments (4 h and 2 days), we performed Welch’s two-sample *t*-tests on log-transformed phytoplasma counts, separately for each treatment group. Statistical analyses were conducted in R [33] using the ‘t.test’ function within a ‘group_modify’ operation from the *dplyr* package [34]. For each treatment, we extracted the *t*-statistic, degrees of freedom, *P* value, mean log-transformed load for each group and the 95% CI of the mean difference. Data visualization was performed using the *ggplot2* package [35], plotting the mean log-transformed phytoplasma load across treatments.

To answer the research questions, we used the following approaches.

Q1. Formal statistical testing to investigate the effects of factors considered in the experiment was performed using a Bayesian approach as described below. To analyse the effects of *treatment* (2 days versus 4 h), *time* (9 samplings) and insect *part* (head versus body) on phytoplasma load, we used a Bayesian hurdle-lognormal model that accounts for zero-inflated load data while incorporating individual-level variation in body part effects. The hurdle-lognormal model is designed to handle data in which many observations are zero, but when a non-zero value does occur, it follows a continuous distribution. The model splits the analysis into two parts: [1] a presence–absence model using a Bernoulli process, where each outcome is either zero (phytoplasma not detected) or non-zero (phytoplasma detected), with the probability of detection affected by the explanatory variables (*treatment*, *time*, *part*); and [2] a phytoplasma load model that models the continuous non-zero values only.

The following hierarchical model was specified: log (phytoplasma load) ∼ treatment+time+part+(1 ∣ individual:part)

where the fixed effect *treatment* had two levels (2 days and 4 h), *time* had nine levels representing consecutive time periods and *part* had two levels (head and body).

The random effect represents individual-level variation in body *part* effects, with a nested *individual:part* random intercept that accounts for variation in part-specific phytoplasma load across individuals. The response variable (phytoplasma load) was log-transformed to handle right-skewed data and to reduce heteroscedasticity.

The decision to include *part* as both a fixed and random effect, and to consider *sex* as an additional fixed effect, was based on a series of model comparisons, starting from a simpler model that included *part* only as a fixed effect and proceeding with different model structures. The model excluding sex and including the (1 | *individual:part*) random effect (see formula above) showed superior predictive performance and was therefore retained as the final model. Leave-one-out cross-validation was used to test the predictive ability of competing model structures [36].

The model included weakly informative priors to ensure reasonable shrinkage with fixed effect coefficients *β*~*N* (0,5), intercept *α*~*N* (0,5) and random effect sds *σb*~*Cauchy* (0,1). The model was implemented in *brms* using four Markov *chain Monte Carlo* chains, each with 2,000 iterations (1,000 warm-up), with adaptive Hamiltonian Monte Carlo adapt_delta=0.99 to improve sampling efficiency.

The analyses were carried out using the *brms* package [37] and graphed using *ggplot* in R.

**Fig. 1.**
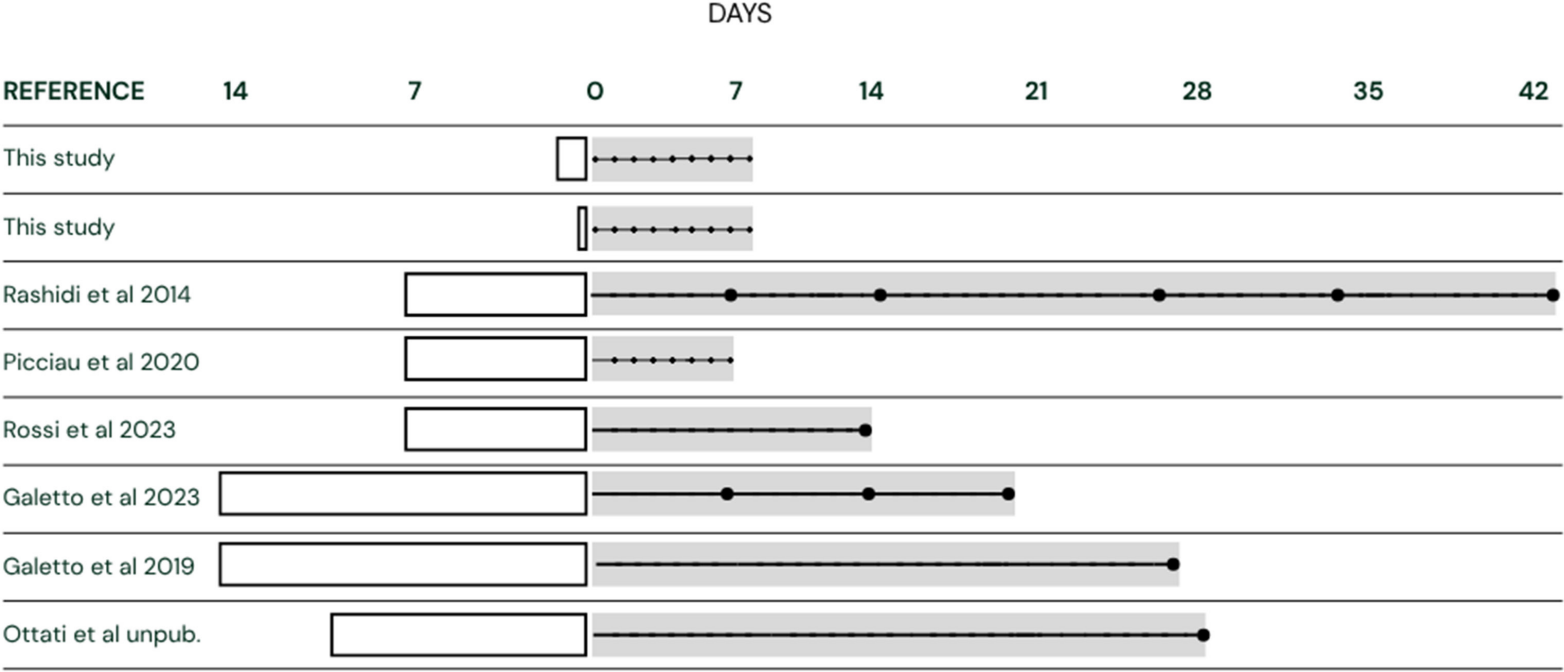
Summary of the published empirical experiments compared to the methodological conditions of this study carried out using the biological model *E. variegatus*–FD-Dp. White boxes before the zero (0) represent AAP length (days); grey boxes represent post-acquisition period length; black dots indicate sampling times, i.e. when specimens were sampled and phytoplasma load measured. Data from Ottati *et al.* are unpublished.

Q2. To model phytoplasma load dynamics over time beyond the 8 days of this study, we compiled a dataset combining our original experimental data (4 h and 2 days acquisition durations, sampled every 16 h for 8 days) with literature-derived observations from longer AAPs (7–14 days) and variable post-acquisition intervals. The dataset included metadata on acquisition time (*treatment*, in days), sampling time (*time*, in days) and tissue type (*part*: head or body). A summary of data acquired from selected literature is provided in Fig. 1. To account for heterogeneous data from Rashidi *et al.* [8] and Picciau *et al.* [18], which provide the average phytoplasma load per treatment per time, a weight variable (weights) was applied to each observation. For the other metadata, data per individual were provided. Phytoplasma load was expressed as GU ng⁻¹ insect body DNA and was log-transformed. We fit a generalized additive model (GAM) to the combined dataset using the *mgcv* package [38] in R [log_value~s(*time*, *k*=7)+s(*treatmen*t, *k*=5)+part]. This model included smooth terms (s) for both sampling time and acquisition duration and a fixed effect for tissue type (*part*). Model predictions and CIs were used to generate time-dependent phytoplasma load trajectories for each acquisition duration and tissue.

Q3. Lastly, to assess whether phytoplasma load increases sufficiently over time under different AAP treatments to approach the threshold titre for vector competence (10^3^–10^4^ GU ng⁻¹ insect DNA) and the estimated carrying capacity (~6×10⁵ GU ng⁻¹ insect DNA), or whether shorter AAPs limit the phytoplasma’s ability to establish long-term colonization, we evaluated the phytoplasma multiplication rate per day during the post-acquisition period, using our data and published data from Picciau *et al.* [18], Rashidi *et al.* [8] and Galetto *et al.* [19], with AAPs ranging from 7 to 14 days (Fig. 1). We adopted a hybrid analytical approach combining both model-based smoothing and direct empirical data calculations. For each experiment and treatment, we fitted GAMs to the log-transformed phytoplasma load data as a function of time. This flexible smoothing method accounts for non-linear trends and irregular sampling intervals. From the fitted GAM, we predicted phytoplasma loads at the start and end of the time interval and calculated the average daily multiplication rate as the geometric mean growth rate. CIs were estimated using both the delta method and bootstrap resampling. CIs from the delta method were obtained by propagating the standard errors of GAM predictions at the start and end of the interval on the log₁₀ scale, assuming approximate normality, and back-transforming these uncertainties through the geometric growth-rate formulation to derive 95% confidence bounds. In parallel, uncertainty was assessed using non-parametric bootstrap resampling by repeatedly resampling observations with replacement, refitting the GAM and recalculating multiplication rates. Bootstrap-based 95% CIs were defined by the 2.5th and 97.5th percentiles of the resulting rate distribution (1,000 iterations). Additionally, multiplication rates were calculated directly from observed log-transformed phytoplasma loads at the start and end time points without smoothing. This empirical approach provides a straightforward estimate of net growth over the interval and serves as a benchmark for comparison against GAM-based estimates. GAM-based smoothing captures complex temporal patterns and reduces noise inherent in biological data, potentially yielding more robust multiplication rate estimates, especially when sampling is sparse or irregular. However, empirical calculations preserve empirical growth signals without assumptions imposed by smoothing, providing an intuitive reference point. By comparing the two methods across all datasets, we ensured that our conclusions on phytoplasma growth dynamics are consistent and not artefacts of modelling choices. Discrepancies between methods were examined through CI overlaps and correlation analyses. We summarized multiplication rates and CIs for each experiment and AAP treatment. Differences between GAM and empirical estimates were assessed, and their correlation was examined to validate robustness. Finally, multiplication rates were interpreted in the context of the phytoplasma’s known minimum threshold titre for vector competence (10^3^–10^4^ GU ng⁻¹ insect DNA, Material S1) and carrying capacity (10⁵ GU ng⁻¹ insect DNA, Material S1) to evaluate whether longer AAPs facilitate colonization near this biological limit, while shorter AAPs constrain it.

## Results

### Spatio-temporal phytoplasma accumulation under short acquisition conditions (Q1)

On average, phytoplasma load was significantly lower in females (mean±se: 0.218±0.07) than in males (0.642±0.095) for the 2-day acquisition treatment (*t*=−3.55, *df*=156, *P*<0.001, 95% CI [−0.66, −0.19]). For the 4-h acquisition treatment, phytoplasma load was slightly higher in females (0.283±0.10) than in males (0.188±0.06), but this difference was not significant (*t*=0.79, *df*=95.7, *P*=0.431, 95% CI [−0.14, 0.33]).

When considering the body part, phytoplasma load was significantly higher in the body (0.73±0.10) than in the head (0.13±0.05) for both the 2-day acquisition (*t*=5.29, *df*=124, *P*<0.001, 95% CI [0.37, 0.82]) and the 4-h acquisition treatment (body, 0.38±0.10; head, 0.07±0.03; *t*=2.82, *df*=78, *P*=0.006, 95% CI [0.09, 0.53]).

**Fig. 2.**
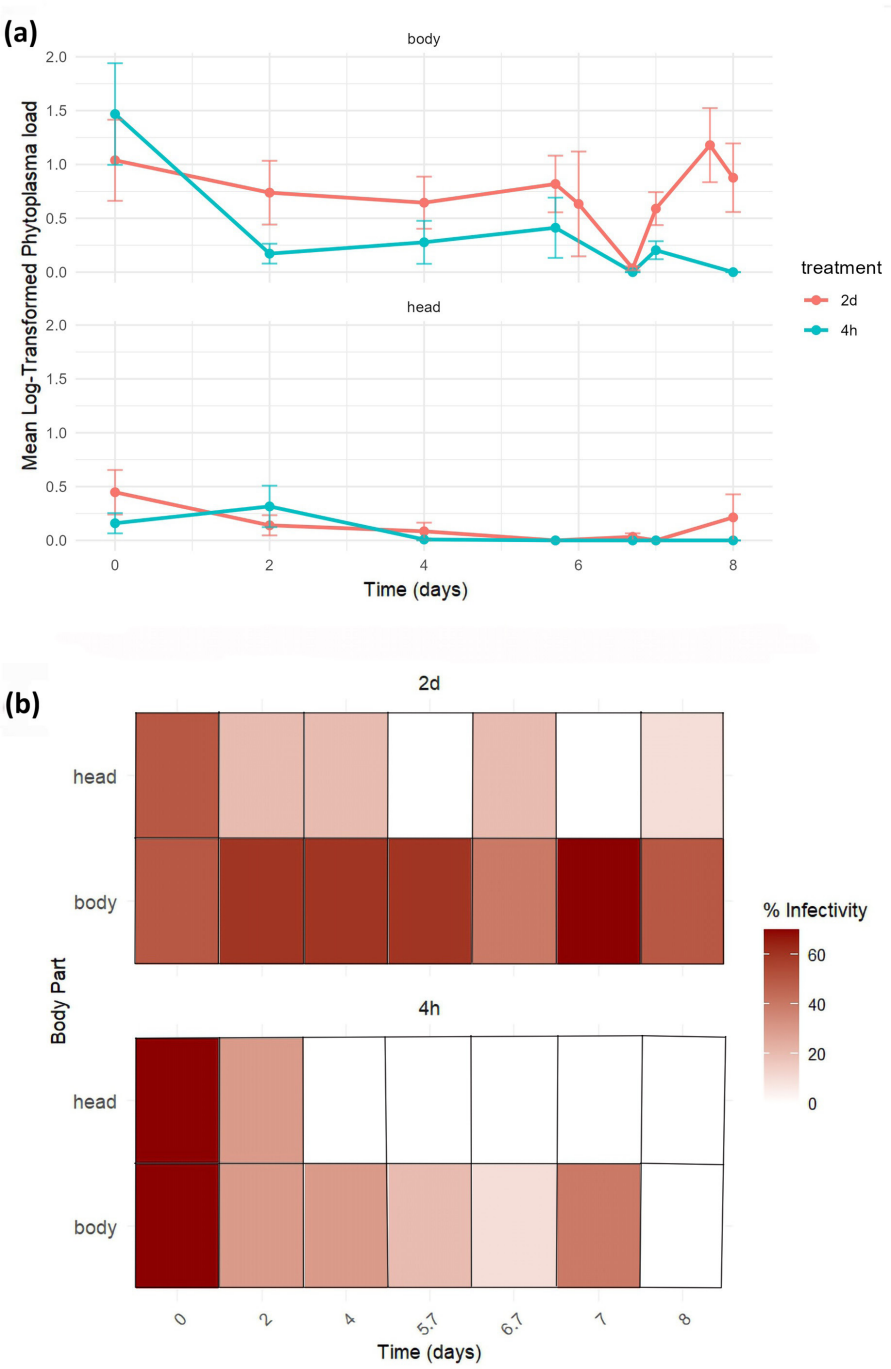
(**a**) Mean log-transformed phytoplasma load in individual insects over time, measured as dpa. Results are shown separately for the body (top panel) and the head (bottom panel), with two acquisition time treatments: 2 days (red) and 4 h (blue). Each point represents the mean phytoplasma load at each time point, highlighting temporal trends and differences between body part and treatments. (**b**) Percentage of infected individuals across head and body regions over time, under two acquisition time treatments: 2 days and 4 h. The top panel depicts progression under the 2-day treatment, while the bottom shows results from the 4-h treatment. Darker shades represent higher infection rates across both body parts.

Regardless of acquisition treatment, the mean log-transformed phytoplasma load in the insect body declined steadily until 6.7 days post-treatment and then began to increase (Fig. 2a). In the head, phytoplasma loads were consistently lower than in the body and showed little variation between 4 and 7.7 days post-treatment, followed by an increase after day 7 in the 2 days AAP.

When considering the percentage of individual heads and bodies infected over time, individuals that underwent the 2-day acquisition maintained a moderate level of infection consistently across the recorded time points and the body displayed higher infectivity than the head, remaining relatively stable throughout. The darker colour bands in the body row highlight persistently elevated infection percentages (Fig. 2b, top panel). For the individuals subjected to 4-h acquisition, initial infection levels were high in both body parts; however, a more rapid decline was observed in the head, indicating transient infectivity, whereas infection in the body decreased gradually (Fig. 2b, bottom panel). The sharper temporal gradient in the head region suggests that this contribution may be due to phytoplasma cells still present in the first tract of the stylet in the first two periods. Phytoplasmas never reached the salivary glands until our last sampling date (8 dpa).

**Fig. 3.**
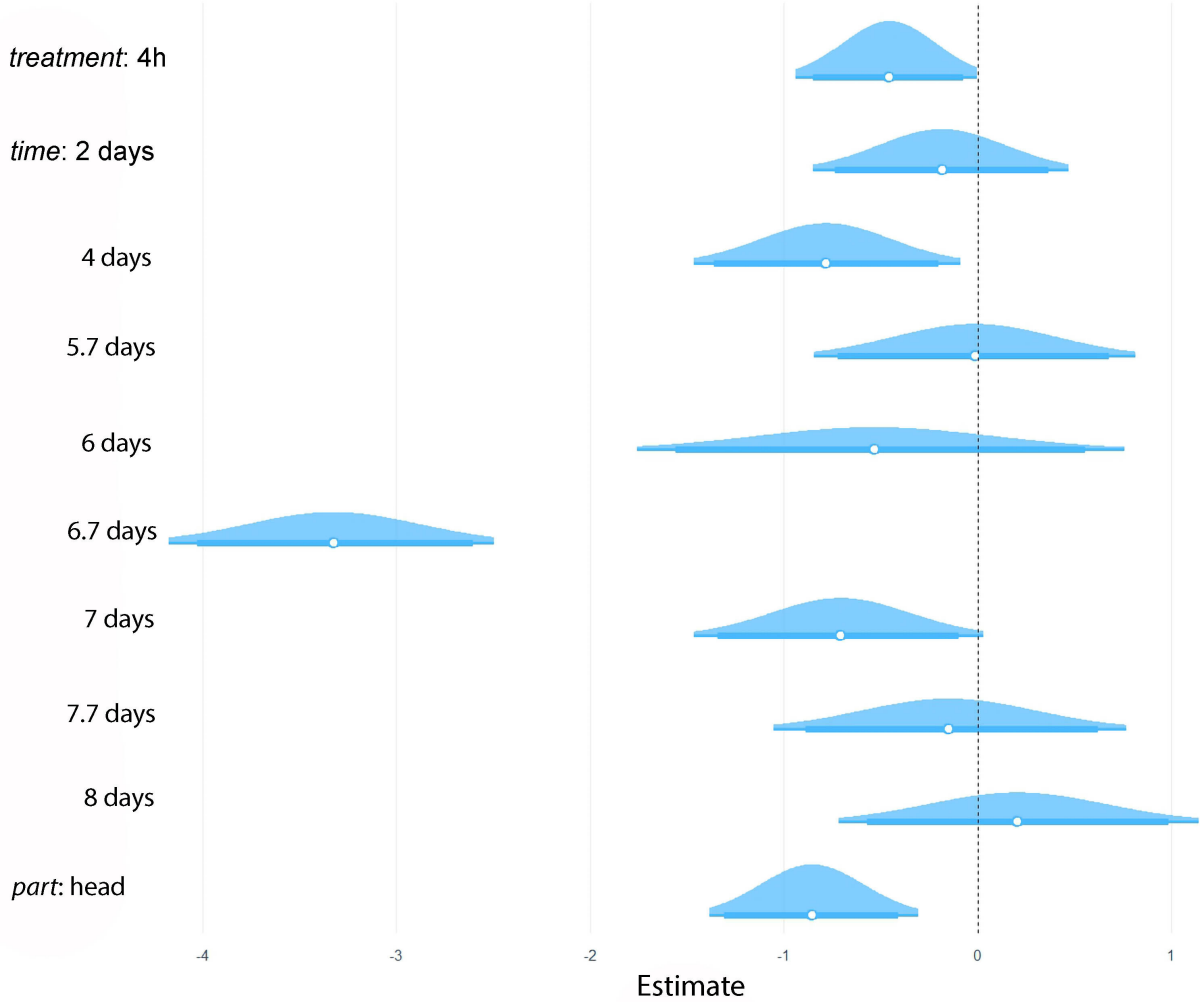
Posterior distributions of fixed effects from the Bayesian hurdle-lognormal model predicting log-transformed phytoplasma load in insect tissues (*n*=292). The model includes acquisition duration (treatment: 2 days versus 4 h), post-acquisition time points (days) and tissue type (body versus head) as fixed effects. Distributions represent posterior means with 90% (thicker) and 95% (thinner) credible intervals. Baseline conditions are 2-day acquisition time (treatment), 0 dpa (time) and body (part).

To evaluate how phytoplasma load varies over time, across two main body parts, and at different times of acquisition on infected plants, we fitted a Bayesian hurdle-lognormal model with individual nested within body part as a random effect (*n*=292). The model accounted for the high proportion of zero counts by separating the occurrence (hurdle) from the magnitude of non-zero pathogen loads. The posterior mean of the intercept was 0.47 (95% CI: −0.06–1.03), representing the expected log-phytoplasma count under baseline conditions (2-day acquisition time, 0 dpa and body part). The 4-h acquisition treatment resulted in significantly reduced phytoplasma load (estimate: −0.46, 95% CI: −0.94 to −0.00), and the head was also associated with lower phytoplasma load compared to the body (estimate: −0.85, 95% CI: −1.38 to −0.31) (Fig. 3). Temporal variation was evident, with phytoplasma load dropping sharply after 6.7 dpa (estimate: −3.32, 95% CI: −4.17 to −2.49), suggesting a possible bottleneck or clearance event. Earlier and later time points did not show consistent trends, and most credible intervals overlapped zero. The hurdle component estimate (hu=0.69, 95% CI: 0.63–0.74) indicates a moderate proportion of zeros across the dataset. Model diagnostics (Rhat ≈ 1, effective sample sizes>1,000 for most parameters) suggest convergence and good mixing, though random effects [sd (Intercept), sigma] had higher uncertainty.

### Long-term predictions of phytoplasma dynamics from short acquisition events (Q2)

**Fig. 4.**
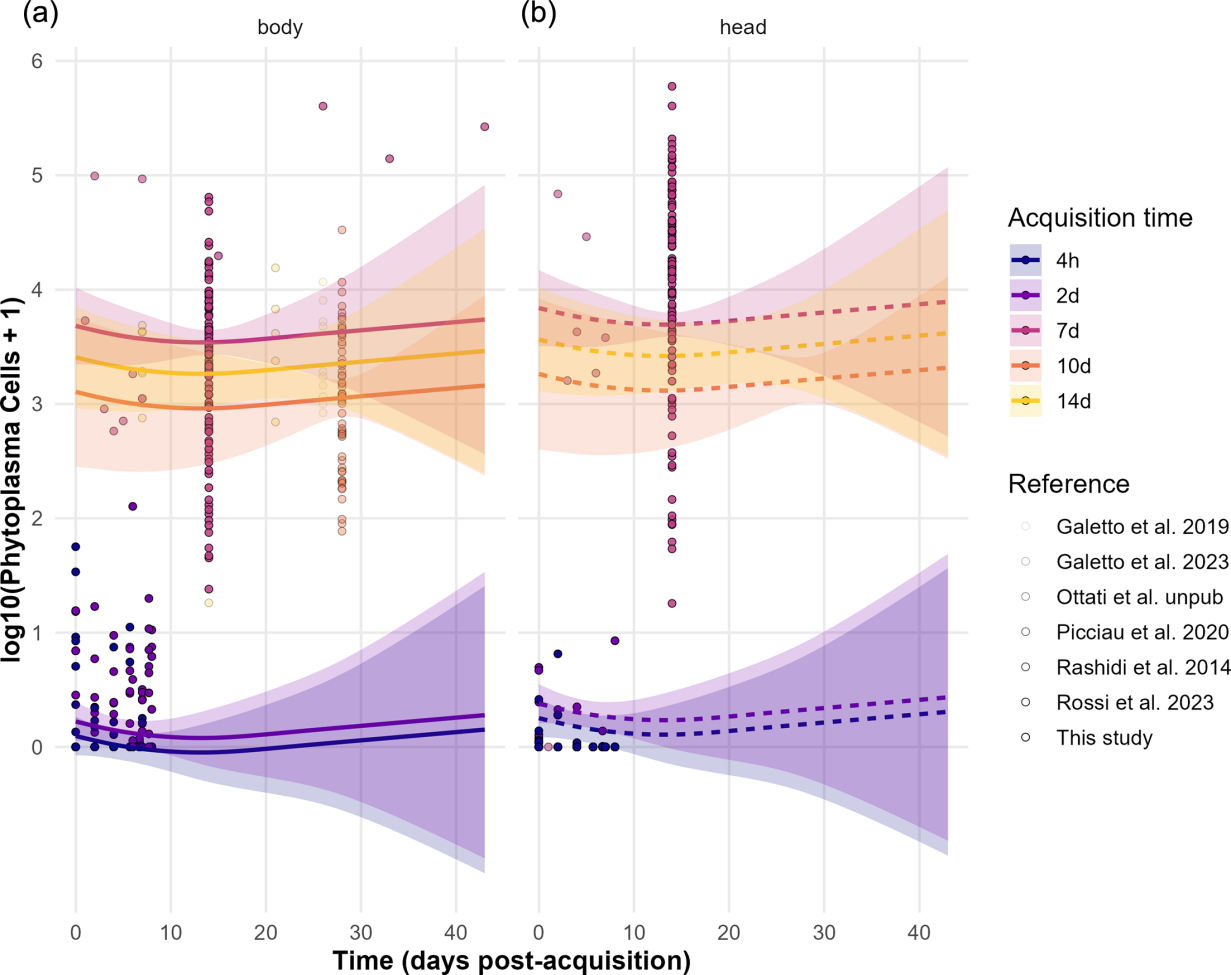
Predicted and observed phytoplasma load dynamics across host tissues and acquisition times. Phytoplasma load (log₁₀-transformed cell counts+1) is plotted over time (dpa) for (a) body and (b) head tissues. Coloured ribbons and solid lines represent predictions and 95% CIs from a GAM fitted across AAPs of 4 h, 2 days, 7 days, 10 days and 14 days. Observed data points are overlaid, coloured by acquisition time, with border thickness indicating reference type (experiments from the literature). The model predicts limited phytoplasma accumulation for minimal acquisition durations (4 h and 2 days, this study), with higher loads and increasing trends in individuals exposed for longer AAPs. Patterns are consistent across tissues, though accumulation is generally lower in head tissue. Literature-based data were integrated with appropriate weights to account for variation in data resolution.

Phytoplasma load (GU ng⁻¹ insect DNA), measured as log₁₀ (Phytoplasma load+1), varied markedly over time depending on both the tissue type (head versus body) and the duration of the AAP on infected source plants (Fig. 4). Predicted curves based on a GAM, combining data from the literature (Fig. 4, upper part of the graphs) and our experimental data (Fig. 4, lower part of the graphs) revealed distinct temporal trends across acquisition treatments.

For individuals with prolonged exposure to infected plants (7, 10 or 14 days, primarily from the literature), phytoplasma load increased consistently over time in both body (Fig. 4a) and head (Fig. 4b) tissues. In contrast, individuals exposed for only 4 h or 2 days (data from this study) showed substantially lower phytoplasma loads during the same post-acquisition period. However, rather than declining, the predicted curves for these short AAPs follow the same trend as for the prolonged AAPs, suggesting that phytoplasmas may persist at low levels without immediate clearance. Importantly, the inclusion of data from the literature [8,18,21, 39] in the model provided a broader temporal context, capturing phytoplasma trajectories up to 42 dpa.

### Multiplication rates and proximity to phytoplasma threshold value and carrying capacity (Q3)

Phytoplasma multiplication rates per day were estimated for five experimental conditions representing varying AAPs, using two complementary approaches: GAMs fitted to log-transformed phytoplasma loads and direct empirical data calculations based on start and end time points (Table 1).

**Table 1.** Phytoplasma multiplication rates per day under different AAPs for this study (4 h and 2 days) and other experiments from the literature (7 and 14 days), estimated using GAMs and empirical calculations, with 95% CIs

Source of data	AAP (days)	Rate per day (GAM)	95% **CI** (GAM bootstrap)	Rate per day (empirical)	95% CI (empirical)
This study	0.167	0.93	0.86–1.02	0.83	0.74–0.92
This study	2	0.93	0.86–1.02	0.98	0.87–1.09
Picciau *et al.* 2020	7	1.00	0.48–1.42	1.61	0.70–2.09
Rashidi *et al.* 2014	7	1.40	0.98–1.50	1.16	0.96–1.17
Galetto *et al.* 2023	14	1.03	0.95–1.26	1.03	0.96–1.11

**Fig. 5.**
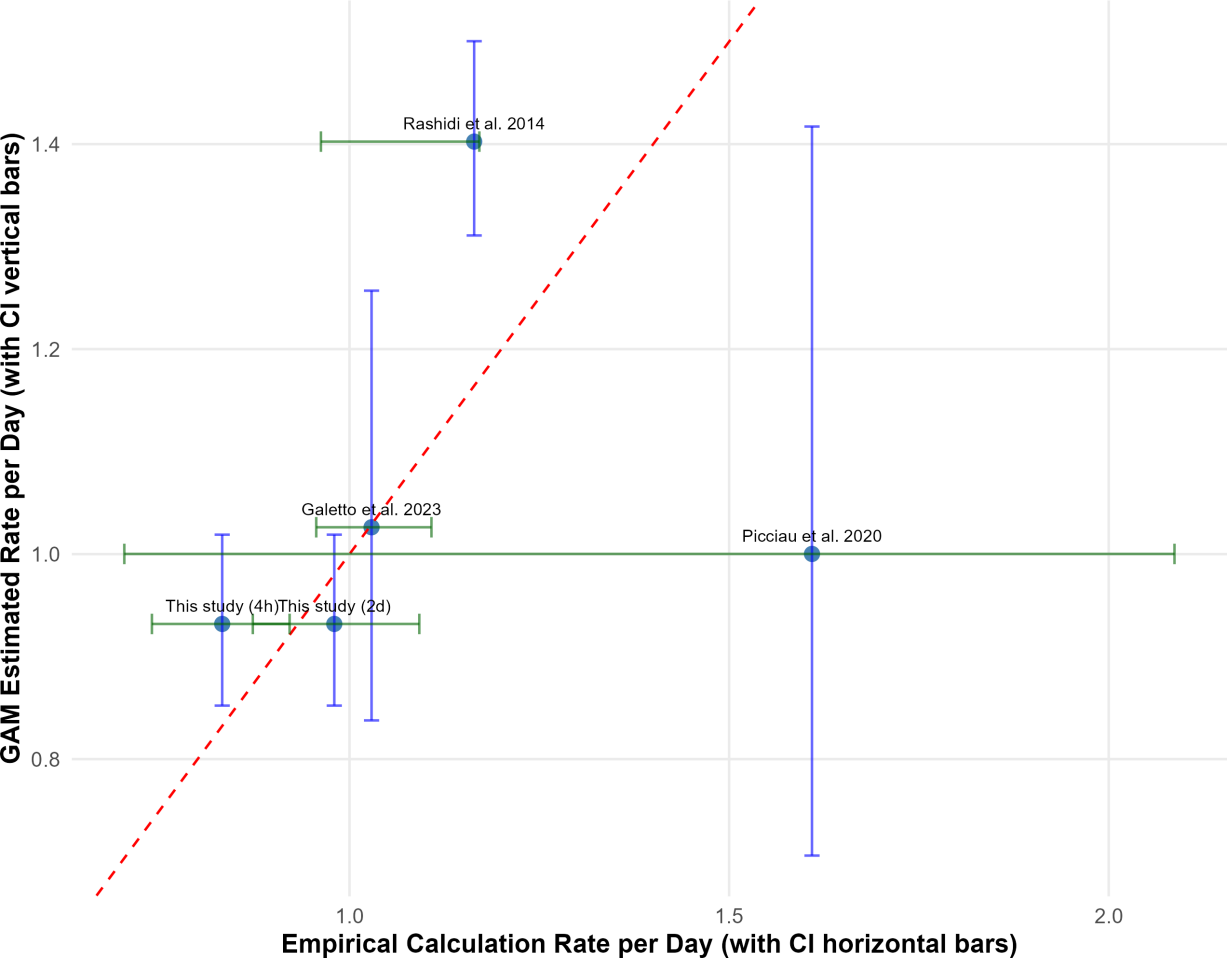
Comparison of empirically calculated and GAM-predicted multiplication rates, with 95% CIs. A dashed line indicates deviation from perfect agreement between the two measures.

The multiplication rates calculated for the experiments in this study are slightly below 1 for both AAP treatments, indicating that the phytoplasma load tends to decrease over time or stay constant and is unlikely to reach the empirical competence threshold titre (10^3^–10^4^ GU ng⁻¹ insect DNA) and carrying capacity (6×10⁵ GU ng⁻¹ insect DNA). Although mean multiplication rates for both 4-h and 2-day AAPs were slightly below 1, the upper bounds of the GAM-derived CIs (up to 1.02 for both the 4-h and 2-day AAPs) and empirical CIs (up to 1.09 for the 2-day AAP) indicate that positive growth may occur in a subset of individuals, suggesting potential heterogeneity in phytoplasma multiplication dynamics. A multiplication rate equal to 1, as observed in Galetto *et al.* [19] for both empirical and GAM-predicted, suggests very slow growth, requiring a prolonged period to approach the carrying capacity (~ 311 days, Table S2). However, the wide CI around this estimate reflects considerable uncertainty in the model. When the multiplication rate exceeds 1, the time required to reach the competence threshold titre decreases dramatically. For example, based on empirical data from Picciau *et al.* [18], the threshold titre could be reached in ~9 days, whereas 19 days are needed to reach carrying capacity (Table S2). Nonetheless, the broad CIs for both the GAM model and the empirical data highlight the need for caution in interpretation, as these may reflect individual variability or limited sampling time points (Fig. 5). The multiplication rates derived from both methods reveal a consistent trend of increasing phytoplasma growth with longer AAPs.

## Discussion

### Comparison of 4-h and 2-day AAP spatio-temporal dynamics during the LP (Q1)

Phytoplasma spread within insect vector tissues and multiplication in host cells have been studied extensively to determine the infection status of the vector and its competence [40,41]. The spatio-temporal dynamics of phytoplasma multiplication within insect vector tissues have been investigated using various approaches, including transmission electron microscopy, immunohistochemistry-based three-dimensional (3D) imaging, whole-mount immunofluorescence techniques, real-time qPCR and ELISA [8,17, 40, 42,45]. To ensure a sufficient initial phytoplasma load and colonization of all organs, these studies have primarily focused on observations made after the minimum LP (7 dpa) and typically allowed insects to acquire phytoplasmas for 7 or more days [8,17, 40, 42, 44, 45]. This focus on prolonged AAPs has left a gap in our understanding of the earliest stages of phytoplasma infection and colonization of insect tissues. In particular, it remains unclear whether a very short acquisition period is sufficient to allow phytoplasmas to fully colonize the vector, render it infectious and reach carrying capacity. Clarifying the dynamics of phytoplasma multiplication during the early post-acquisition phase may provide insights into the potential epidemiological consequences of incidental acquisition events in the field. Because phytoplasmas may manipulate the phenotypes of infected plants in ways that attract both vector and non-vector insects, this may sometimes lead to incidental acquisition of phytoplasmas by new potential vectors and, potentially, result in increased phytoplasma spread through the environment.

Our experimental tests of two short AAPs, 4 h (simulating an incidental acquisition event) and the 2 days established minimum, allowed us to evaluate the factors that constrain phytoplasma multiplication in the early stage of colonization (LP), considering sex, body part and time after acquisition. The factors considered in our study have been shown to be fundamental and indirectly affect the infection status and the population dynamics of different bacteria within insect hosts [46,48]. In our study, phytoplasma load was significantly lower in females than in males under the 2-day acquisition treatment, while no significant differences were observed in insects exposed to a 4-h AAP. This suggests that sex has an effect under longer AAP, with differences increasing over time, in line with previous results on the same *E. variegatus*–FDp patho-system [48]. However, including sex as a factor in our Bayesian model decreased model performance, suggesting that sex is not a key determinant of phytoplasma multiplication dynamics in the insect body during the early stages of colonization. Overall, longer acquisition periods appear to stabilize phytoplasma colonization, consistent with sex-related differences reported in the literature.

Our experiments showed no phytoplasma multiplication during early stages of infection but a steady decrease (99% on average) in phytoplasma load until 6.7 days, when the phytoplasma cells, likely localized extracellularly, may be cleared from the gut lumen, possibly reflecting an immune response by the vector [49,50]. Between 7 and 8 days, we documented an initial multiplication in the midgut, reflected by consistently higher phytoplasma load in the body compared to the head across both acquisition periods. The body showed more stable infection dynamics over time, while the head exhibited transient and variable loads, consistent with previous results based on 7-day AAPs. This shows that phytoplasmas cross the anterior midgut epithelium and multiply after about 7 dpa, reaching an abundance 10-fold higher after 28 dpa, consistent with the results by Koinuma *et al.* [17]. However, our results further suggest a strong AAP-dependent effect on multiplication during the early stage of infection, suggesting that a higher ingested phytoplasma load may accelerate the colonization of cells and other organs (Fig. 2) as previously observed for ‘*Ca*. L. solanacearum’ and the psyllid vector *Bactericera cockerelli* (Šulc) [51].

### Predicting phytoplasma load dynamics: short versus long acquisition periods (Q2)

By modelling phytoplasma load trajectories using new and previously published data, we were able to assess whether and how infection dynamics vary within and beyond our tested AAP durations. Our prediction over a period of 42 dpa suggests that when vectors are exposed to infected plants for 7, 10 or 14 days, phytoplasmas accumulate, post-acquisition, at rates consistent with effective colonization and phytoplasma multiplication. Interestingly, although reducing exposure to 4 h or 2 days (data from this study) yields substantially lower phytoplasma load, we observed the same post-acquisition found in insects exposed to longer AAPs. Although, in the head, predicted loads remained close to baseline across time, indicating a limited or delayed ability of phytoplasma to move into or multiply within this compartment after minimal acquisition periods, the predicted curves for these shorter AAPs did not decline but instead followed a similar trend to those of the prolonged AAPs (Fig. 4). This suggests that phytoplasmas acquired during short-duration feeding events may persist at low levels without undergoing immediate clearance. This raises the possibility that, under favourable conditions and extended post-acquisition time, phytoplasmas thus acquired could eventually accumulate, although our model suggests that any such increase would remain well below the levels observed after longer AAPs. A major constraint on systemic colonization and multiplication of a pathogen within its vector may be the vector’s lifespan and the age-dependent response to infection, which sets an upper limit for how long the pathogen can persist, multiply and potentially be transmitted [52,53]. For our experimental vector, *E. variegatus*, lifespan varies from a maximum of 62 days to an average of 41 days, depending on whether individuals are infected with entomopathogens [54]. Moreover, several authors [47,49, 55] reported a significant reduction of longevity induced by FDp on its experimental and natural vectors, *E. variegatus* and *S. titanus*, although the host plant can affect the magnitude of the impact.

The response of the vector’s immune system to the phytoplasma can also affect infection dynamics to varying degrees [50]. Some phytoplasmas may suppress immunity to facilitate colonization [56], while others induce defences that limit infection [49]. In our study, the decrease in phytoplasma load during the first 7 days likely resulted from the insect’s immune response [49].

The divergence in predicted infection trajectories between short and long AAPs suggests a biological threshold between 2 and 7 days of exposure, below which phytoplasma colonization may not succeed. Nevertheless, in some other comparable vector–pathogen models, very short minimum acquisition periods ranging from 30 min to 24 h [57,58] are sufficient to allow transmission. This has key implications for vector competence and transmission efficiency in natural systems, as even short feeding events may result in persistent low-level infections with uncertain outcomes over longer timescales.

Because we lack long-term post-acquisition data for the shorter AAPs used in our study, the long-term trajectories for short AAPs extrapolated based on patterns observed in longer AAP experiments and by comparable multiplication rates should be taken with caution.

### Shorter AAPs may be sufficient to reach threshold titre for vector competence but not sufficient to reach carrying capacity (Q3)

Phytoplasmas are recalcitrant bacteria and their biological parameters are very difficult to measure under experimental conditions; thus, generalized observational studies need to be interpreted with caution. Establishing controlled experiments on uncultured bacteria within living hosts (vectors or plants) involves managing a number of variables. Moreover, extrapolating assumptions derived from controlled experimental systems to natural environments can be challenging and, at times, misleading [59]. Multiplication rate is one of the most complex parameters to model for fastidious, not-yet-cultivable bacteria, for which no direct empirical estimates currently exist. Studies on cultured bacteria have shown that multiple host-related factors, such as physiological condition and developmental stage, modulate multiplication rate [60,62]. Such variability may influence whether the minimum threshold required for vector transmission is reached [63]. Thus, this transmission threshold may also vary depending on the specific vector–pathogen combination and the vector’s life stage.

Using specimens collected from the wild with unknown AAPs, Jarausch *et al.* [24] showed that the minimum inoculation titre (i.e. the lowest phytoplasma titre observed in individual vectors that successfully transmitted the phytoplasma) was ~3.5 times lower for *Allygus* spp. (62 GU ng⁻¹ insect DNA) than for *Orientus ishidae* (Matsumura) (218 GU ng⁻¹ DNA); two leafhopper vectors are able to transmit FD from alder to alder or to grapevine, with the latter species also reaching significantly higher phytoplasma loads (10³–10⁴ GU ng⁻¹ DNA) when maintained in captivity. Using the *E. variegatus*–FD-Dp system in an experimental setting, Picciau *et al.* [18] estimated the minimum phytoplasma load in the insect body required for inoculation after a 7-day AAP. This threshold, determined at 4 dpa, averaged between 10³ and 10⁴ GU ng⁻¹ DNA. Moreover, using a different phytoplasma (CYp), Galetto *et al.* [25] showed that *E. variegatus* successfully transmitting CYp carried significantly higher pathogen loads than non-transmitters, both at the whole-insect and body part levels. Indeed, only individuals with sufficiently high titres, particularly in the head, served as transmitters (10³–10⁴ GU ng⁻¹ DNA), whereas non-transmitters harboured sub-threshold concentrations (10^2^–10^3^ GU ng⁻¹ DNA). The authors proposed that the low titre of CYp in non-transmitters may result from the inability of the phytoplasma to overcome barriers at the midgut or salivary gland level, preventing full colonization of the body. In the *Ricania speculum*–FD-Dp system [20], the highest phytoplasma loads, averaging 10^1^ GU ng⁻¹ DNA in the body and 10^2^ GU ng⁻¹ DNA in the salivary glands, were insufficient to allow inoculation of phytoplasma into *Vitis vinifera* and *Clematis vitalba*. In the case of phytoplasmas, the multiplication rate and the minimum threshold titre required for successful transmission depend on the specific patho-system [18,20, 24, 25] and are, in turn, influenced by the evolutionary history of the association [64]. Over time, phytoplasmas and their potential vectors are expected to influence each other’s evolution through the interplay between molecular-level interactions and the vector’s immune response, which may drive the transition from a parasitic to a commensal association and vice-versa.

Determining this minimum threshold and carrying capacity of a population is most accurately achieved through an integrative approach combining mathematical modelling, controlled experimentation and time-series analysis [26]. Indeed, previous attempts to measure carrying capacity in bacterial model systems such as *Escherichia coli* [65,67] combined empirical data with logistic models. Carrying capacity, a crucial concept in population biology, has rarely been explicitly considered in vector-borne pathogens such as phytoplasmas. Rossi *et al.* [21] suggested that lower phytoplasma loads in mixed-strain infections compared to single-strain infections may indicate competition between FD-C and FD-D phytoplasma strains, possibly limited by the vector’s carrying capacity. Other studies calculated the carrying capacity of an overall population of phytoplasmas in a specific environment, considering the sum of cells from a population in a certain association of insect and plant hosts [68,69]. We adopted a multifaceted strategy that integrates empirical and modelling methodologies to provide the most reliable approximation of the spatio-temporal dynamics of phytoplasma multiplication and population density within its insect vector. Furthermore, we contextualized these findings in relation to the vector’s potential lifespan, thereby offering deeper insights into critical aspects of phytoplasmosis epidemiology and, more specifically, the possible outcomes of incidental feeding events in nature. We estimated phytoplasma multiplication rate during the early stage of colonization in the insect body when the minimum titre was very low (1–10 GU ng⁻¹ DNA for 4-h and 2-day AAPs). We also calculated multiplication rates based on published data, where maximum values of 10^3^–10^4^ GU ng⁻¹ DNA were recorded for longer AAPs (7–14 days). Thus, we sought to estimate the phytoplasma multiplication rate using a dual and complementary approach, combining GAMs with empirical calculations. When the multiplication rate is only slightly above 1, the minimum threshold can be reached within a time frame reasonably compatible with the lifespan of the insect vector. As both initial concentration and multiplication rate increase, the time required drops dramatically (Table S2). The classical bacterial growth cycle comprises different phases, and while multiplication rate is traditionally linked to nutrient availability and environmental conditions, it is now also understood to vary with the initial concentration, reflecting a population-level rather than an individual cell property [70,72]. Taken together, these observations indicate that, although the initial phytoplasma concentration within the vector is related to AAP length, it subsequently changes over time and thereby modulates the multiplication rate dynamically throughout the course of infection.

## Conclusion

Our findings demonstrate that very short AAPs, potentially resulting from incidental or brief feeding events on infected plants, consistently lead to lower phytoplasma loads after an 8-day LP compared to prolonged feeding events lasting up to 2 days. These results suggest that the duration of vector feeding plays a critical role in determining the efficiency of phytoplasma acquisition during the early stage of colonization and subsequent phytoplasma multiplication.

Our modelling approach, combining data from several prior studies with new experimental results, enabled the evaluation of projected phytoplasma load over a 40-day period following acquisition. Our results indicate that short AAPs (4 h and 2 days), potentially resulting from incidental or brief feeding events on infected plants, generally yield phytoplasma multiplication rates below unity, leading to stable or declining pathogen loads at the population level. Under these conditions, insects are unlikely to reach the empirical competence threshold or carrying capacity within biologically realistic time frames. However, the CIs associated with GAM-derived multiplication rates and empirical calculations, particularly the upper bounds for the 4-h and 2-day AAPs, indicate that positive growth may occur in a subset of individuals. This variability may reflect individual differences in initial acquisition dose, physiological condition or host–pathogen compatibility. Consequently, while short AAPs appear insufficient to sustain pathogen multiplication at the population level, they may nonetheless allow some individuals to transiently approach the minimum transmission threshold. The overall load remains consistently lower in insects exposed to short AAPs, reinforcing the importance of feeding duration in shaping phytoplasma dynamics.

Based on both empirical and inferred multiplication rates, our results are consistent with prior observations made using cultivated bacteria regarding multiplication dynamics. Our findings suggest that changes in phytoplasma load over time depend in part on the initial concentration acquired during AAPs, but that even short AAPs have the potential to yield loads sufficiently high to facilitate transmission. This has important implications for disease transmission risk in the field, especially when incidental feeding events on infected plants occur.

## Supplementary material

10.1099/mic.0.001700 Uncited Supplementary Material 1.

## Abbreviations

AAP: acquisition access period
CTAB: cetyltrimethylammonium bromide
dpa: days post-acquisition
GAM: generalized additive model
GU: genome units
hpa: h post-acquisition
LP: latent period
qPCR: quantitative PCR
