# Supplementary material for: Tracking the early spatio-temporal dynamics of phytoplasma multiplication within its leafhopper vector
Source: Microbiology (Reading). 2026 Apr 29;172(4):001700. doi: 10.1099/mic.0.001700 (PMC13135482; doi:10.1099/mic.0.001700)
Supplement: Uncited Supplementary Material 1. [file mic-172-01700-s001.pdf]

Tracking the early spatio-temporal dynamics of phytoplasma multiplication within its leafhopper vector

Valeria Trivellone[a*], Francesca Canuto[b], Giulia Lucetti[b], Christopher H. Dietrich[a], Luciana Galetto[b], Cristina Marzachì[b]

Supplementary Material

S1 - Systematic review of quantitative PCR studies tracking phytoplasma load dynamics in insect vectors

To inform the experimental design, we conducted a systematic literature review following PRISMA guidelines (Page et al., 2021; Jones, 2023) to identify and synthesize studies that used quantitative qPCR to assess changes in phytoplasma load in insects over time. Bibliographic searches were performed in Scopus, Pubmed and Web of Science databases. The initial search was conducted on 12 September 2024 and was repeated after the first round of manuscript revision to identify any newly published eligible studies. To reduce the risk of publication bias, grey literature, unpublished material, and other non-peer-reviewed sources were identified and included after the primary searches of the three databases. Two additional studies were included consisting of one unpublished study and one known publication not retrieved by the selected search terms (Ottati et al., unpublished; Picciau et al. 2020). To minimize ambiguity associated with individual search terms and to maximize retrieval of relevant studies, we employed a structured search strategy using both single keywords and combinations of them. Two core terms, *"phytoplasma"* and *"insect"*, were combined with three additional companion keywords using the Boolean operator "AND," as detailed below:

phytoplasma AND quantification AND insect

phytoplasma AND qPCR AND insect

phytoplasma AND real-time PCR AND insect

During the initial screening phase, duplicate records identified across the three independent database searches were removed (see table below). The remaining unique records across all three databases resulted in 152 papers being assessed for eligibility. Three further studies not detected by the search were added (full list of papers, https://doi.org/10.13012/B2IDB-6451062_V1).

Database	Records retrieved	Duplicates removed	Records retained
Scopus	142	37	105
PubMed	84	26	58
Web of Science	177	47	130

Studies were first evaluated based on title and abstract when available; however, for the majority of publications, full-text assessment was necessary to extract the relevant quantitative data.

Inclusion criterium is as follows: (1) studies that used quantitative real-time PCR (qPCR) to measure phytoplasma load in insects under controlled experimental conditions using conventional DNA extraction methods.

Exclusion criteria were applied as follows: (0) not complying with inclusion criterium; (1) studies in which phytoplasma quantification was based on normalization using housekeeping reference genes in the absence of a standard curve; (2) studies employing digital droplet PCR (ddPCR); (3) studies using insects that acquired phytoplasmas under field conditions rather than controlled experimental settings; (4) studies reporting phytoplasma quantification only at the sample level (Genome Unit/insect) or with different expression units rather than at the host DNA level (GU/ng insect DNA); (5) studies that did not provide raw quantitative data or sufficient information to derive it; (6) studies reporting phytoplasma quantification based on cDNA rather than genomic DNA; (7) studies that do not focus on *Euscelidius variegatus*-FD system.

A total of six studies were ultimately selected and focused specifically on the *Euscelidius variegatus*–Flavescence dorée phytoplasma model and constituted the core dataset used in the meta-analysis (lines in red, see dataset: https://doi.org/10.13012/B2IDB-6451062_V1). The metadata were scored and recorded (GU/ng insect DNA data per body part per period) in a dedicated database, available upon request from the first author. In addition, eight further studies were retained and examined separately to evaluate vector carrying capacity and to inform the definition of minimum infection or detection thresholds, but were not included in the quantitative meta-analysis (lines in blue, see dataset: https://doi.org/10.13012/B2IDB-6451062_V1)

References

Jones, E.P. (2023). Systematic Review Manuscript Template. University of North Carolina at Chapel Hill (UNC) Health Sciences Library. https://guides.lib.unc.edu/systematic-reviews/write

Page, M.J., McKenzie, J.E., Bossuyt, P.M., Boutron, I., Hoffmann, T.C., Mulrow, C.D., Shamseer, L., Tetzlaff, J.M., Akl, E.A., Brennan, S.E. and Chou, R., 2021. The PRISMA 2020 statement: an updated guideline for reporting systematic reviews. *bmj*, *372*.

S2 - DNA extraction from plant

To determine the infectious status and phytoplasma load in plants to be used as a source of inoculum in the 4h/2day experimental AAP, approximately 0.100 g of leaf tissue was collected from broad bean (*Vicia faba*) exposed to infective insects and ground in liquid nitrogen using a mortar and pestle. Following the evaporation of residual liquid nitrogen, 1 mL of 3% CTAB buffer was added to the powdered tissue, and the mixture was transferred to a 2.2 mL tube. The sample was incubated at 65°C for 20 minutes and subsequently centrifuged at 4,500 rpm for 5 minutes at room temperature. An aliquot of 800 µL of the resulting supernatant was collected and mixed with 800 µL of chloroform:isoamyl alcohol (24:1, v/v), followed by centrifugation for 10 minutes. The aqueous phase was recovered, and 700 µL of isopropanol were added to precipitate the nucleic acids. The resulting pellet was washed with 70% ethanol, dried using a SpeedVac SPD130DLX (Thermo Fisher Scientific), resuspended in 50 µL of 10 mM Tris-HCl buffer (pH 8.0) and analyzed in a Nanodrop ND-1000 spectrophotometer (Thermo Fisher Scientific).

Estimates of host plant phytoplasma loads from other studies ranged as follows: Galetto et al. 2023: 4.7×10^3–5.7×10^5 FDp Genome Units (GU) /ng host DNA; Picciau et al. 2020: 1.2×10^4–2.9×10^5 GU/100 mg plant tissue; Rossi et al. 2023: 4.9×10^4–8.1×10^5 FDp GU/ng of plant DNA.

S3- Nucleic acids extraction - Comparison of approaches

Total DNA and RNA were extracted simultaneously from previously dissected insects in which head and body (thorax + abdomen) were separated. Each dissected portion of the insect was placed in a 1.5 mL tube, frozen in liquid nitrogen and ground using a sterile micro pestle. Subsequently, 350 µL of Lysis Solution, provided in the Spectrum™ Plant Total RNA Kit (Merck) and supplemented with 2-mercaptoethanol (2-ME), was added to the insect tissues. The samples were then incubated at 56°C for 5 minutes and centrifuged at 14,000 rpm for 3 minutes. The resulting supernatant was promptly divided into two aliquots of 150 µL, transferred to a new 1.5 mL tube and used for subsequent nucleic acid extractions (protocols 1 and 2).

In the first procedure (protocol 1), a volume of chloroform was added, then the mixture was centrifuged at 12000 rpm for 20 minutes and 100 µL of supernatant were taken and transferred into a new 1.5 mL tube. Thereafter, 200 µL of isopropanol were added, the precipitated material was collected via centrifugation at 15000 rpm and 4°C for 15 minutes and washed with 300 µL of 70% ethanol. Before drying the pellet using a SpeedVac SPD130DLX (Thermo Fisher Scientific), a 10-minute centrifuge at 15000 rpm and 4°C was performed. Finally, the pellet was resuspended in 15 µL DNase/RNase-free sterile water.

In the second procedure (protocol 2), total DNA and RNA were co-extracted using Protocol A of the Spectrum™ Plant Total RNA Kit, with the filtration step skipped and the protocol further modified slightly as follows: 350 µL of Binding Solution were mixed with supernatant obtained after lysis and centrifugation and loaded onto the supplied binding column. To ensure co-extraction of both DNA and RNA, the DNase digestion step was omitted. Bound nucleic acids were eluted in 30 µL of Elution Solution pre-heated to 60 °C.

In both cases (protocols 1 and 2), nucleic acids quantification was carried out using a Nanodrop ND-1000 spectrophotometer (Thermo Fisher Scientific).

When required, the reverse transcription was carried out on total nucleic acids (averagely 280 ng) with random hexamers and the High-Capacity cDNA Reverse Transcription Kit (Applied Biosystems) following manufacturer's instruction.

RESULTS: A total of 6 individuals, i.e. 12 subsamples (head and body) were extracted either using both protocols 1 and 2 on the same material (4 samples) or only protocol 2 (8 samples). A qPCR experiment compared the Ct (cycle threshold) values between samples extracted with the two procedures (Table S1). For two samples analyzed out four, we found that protocol 2 provided a reliable quantification of phytoplasmas genomic DNA leaving the possibility of an eventual analysis of RNA.

Table S1. Nucleic acid yield and purity and Ct (cycle threshold) resulted from 12 subsamples from insects exposed (FD) or not (NE) to FD phytoplasma, extracted with protocols 1 or 2 and retrotranscribed (Y) or not (N). NA: not amplified.

Insect ID	Sample ID	Sample type	Protocol	Conc. (μg/μl)	260/280	Retro-transcription	Ct
#5_FD	2879	Head	1	17.0	2.31	N	32.9
			2	4.8	1.27	N	NA
						Y	35.0
	2880	Body	1	138.6	1.76	N	35.5
			2	146.1	2.07	N	NA
						Y	33.8
#6_FD	2881	Head	1	-15.0	/	N	NA
			2	19.5	1.36	N	29.4
						Y	30.7
	2882	Body	1	57.0	1.65	N	26.5
			2	117.0	2.06	N	27.6
						Y	24.8
#3_FD	2871	Head	2	29.8	2.03	N	NA
						Y	NA
	2872	Body	2	166.6	2.09	N	NA
						Y	NA
#4_FD	2873	Head	2	37	2.08	N	21.7
						Y	22.4
	2874	Body	2	5.5	0.54	N	19.1
						Y	19.6
#1_NE	2867	Head	2	29.9	2.06	N	NA
						Y	NA
	2868	Body	2	182.6	2.12	N	NA
						Y	NA
#2_NE	2869	Head	2	16.8	1.9	N	NA
						Y	NA
	2870	Body	2	125.1	2.11	N	NA
						Y	NA

Table S2. Time in days (t) for phytoplasma population to reach minimum threshold titer for vector competence (T= 10^3-10^4 GU/ng insect) and carrying capacity (C ≈6 × 10^5 GU/ng insect) in the insect body.

Compared experiments are defined by different AAPs (acquisition access periods), 4 hours (this study, 4h_Tri), 2 days (this study, 2d_Tri), 7 days (7d_Picciau and 7d_Rashidi) and 14 days (14d_Galetto). Multiplication rates (r) and their confidence intervals (CI) are estimated by using two different approaches: generalized additive models (GAMs) and empirical estimates (Emp). Und: undefined.

**Experiment**	**approach**	N_0 [1]	$N(t)$ [1]	**t (lower-upper)**[1]	**r**	**95% CI lower limit**	**95% CI upper limit**
4h_Triv	GAM	10	T= 10^3	-63 (-30; **232**)	0.93	0.86	1.02
			C= 10^5	-126 (-61; **465**)			
4h_Triv	Emp	10	T= 10^3	-24 (-15; -55)	0.83	0.74	0.92
			C= 10^5	-49 (-30; -110)			
2d_Triv	GAM	10	T= 10^3	-63 (-30; **232**)	0.93	0.86	1.02
			C= 10^5	-126 (-61; **465**)			
2d_Triv	Emp	10	T= 10^3	-227 (-33; **53**)	0.98	0.87	1.09
			C= 10^5	-455 (-66; **106**)			
7d_Picciau	GAM	10	T= 10^3	Und (-6; **13**)	1	0.48	1.42
			C= 10^5	Und (-12; **26**)			
7d_Picciau	Emp	10	T= 10^3	**9** (-12; **6**)	1.61	0.7	2.09
			C= 10^5	**19** (-25; **12**)			
7d_Rashidi	GAM	10	T= 10^3	**13** (-227; **11**)	1.4	0.98	1.5
			C= 10^5	**19** (-455; **22**)			
7d_Rashidi	Emp	10	T= 10^3	**31** (-112; **29**)	1.16	0.96	1.17
			C= 10^5	**62** (-225; **58**)			
14d_Galetto	GAM	10	T= 10^3	**155** (-89; **19**)	1.03	0.95	1.26
			C= 10^5	**311** (-179; **39**)			
14d_Galetto	Emp	10	T= 10^3	**155** (-112; **44**)	1.03	0.96	1.11

			C= 10^5	**311** (-225; **88**)			

[1] The time was calculated using the following formula:

$$N(t) = N_0 \times r^t$$

$$t = \frac{\log\left(\frac{N(t)}{N_0}\right)}{\log(r)}$$

where $\boldsymbol{N(t)}$ is the final phytoplasma load, $\boldsymbol{N_0}$ is the initial phytoplasma load, **r** is the multiplication rate and **t** is the time. In bold are the positive values (number of days). When **r** = 1, the phytoplasma load remains constant over time and is therefore equal to $\boldsymbol{N_0}$
